# Photosynthesis, growth, and decay traits in *Sphagnum* – a multispecies comparison

**DOI:** 10.1002/ece3.2119

**Published:** 2016-04-12

**Authors:** Fia Bengtsson, Gustaf Granath, Håkan Rydin

**Affiliations:** ^1^ Department of Plant Ecology and EvolutionEvolutionary Biology CentreUppsala UniversityNorbyvägen 18DSE‐752 36UppsalaSweden; ^2^ Department of EcologySwedish University of Agricultural SciencesBox 7044SE‐750 07UppsalaSweden

**Keywords:** Decomposition, functional traits, peat moss, photosynthetic capacity, production, trade‐offs

## Abstract

Peat mosses (*Sphagnum*) largely govern carbon sequestration in Northern Hemisphere peatlands. We investigated functional traits related to growth and decomposition in *Sphagnum* species. We tested the importance of environment and phylogeny in driving species traits and investigated trade‐offs among them. We selected 15 globally important *Sphagnum* species, representing four sections (subgenera) and a range of peatland habitats. We measured rates of photosynthesis and decomposition in standard laboratory conditions as measures of innate growth and decay potential, and related this to realized growth, production, and decomposition in their natural habitats. In general, we found support for a trade‐off between measures of growth and decomposition. However, the relationships are not strong, with *r* ranging between 0.24 and 0.45 for different measures of growth versus decomposition. Using photosynthetic rate to predict decomposition in standard conditions yielded *R* ^2^ = 0.20. Habitat and section (phylogeny) affected the traits and the trade‐offs. In a wet year, species from sections Cuspidata and Sphagnum had the highest production, but in a dry year, differences among species, sections, and habitats evened out. Cuspidata species in general produced easily decomposable litter, but their decay in the field was hampered, probably due to near‐surface anoxia in their wet habitats. In a principal components analysis, PCA, photosynthetic capacity, production, and laboratory decomposition acted in the same direction. The species were imperfectly clustered according to vegetation type and phylogeny, so that some species clustered with others in the same section, whereas others clustered more clearly with others from similar vegetation types. Our study includes a wider range of species and habitats than previous trait analyses in *Sphagnum* and shows that while the previously described growth–decay trade‐off exists, it is far from perfect. We therefore suggest that our species‐specific trait measures offer opportunities for improvements of peatland ecosystem models. Innate qualities measured in laboratory conditions translate differently to field responses. Most dramatically, fast‐growing species could only realize their potential in a wet year. The same species decompose fast in laboratory, but their decomposition was more retarded in the field than that of other species. These relationships are crucial for understanding the long‐term dynamics of peatland communities.

## Introduction

Species in the bryophyte genus *Sphagnum* are responsible for around 50% of the peat in northern habitats (Turetsky 2003), and although boreal peatlands only cover 2–3% of the earth's land surface, they store about a third of the world soil carbon (Rydin and Jeglum 2013). Not only are they the dominant organism, they also create and maintain these peatlands through peat formation (Rydin et al. 2006). Through acidification and waterlogging, they shape their habitat, thereby making it impossible for most vascular plants and microorganisms to grow because of the anoxic, acidic, nutrient‐poor environment (Rydin et al. 2006). The slow decay of *Sphagnum* species is a key to their success, because it makes waterlogging and carbon sequestration possible. These processes are related to multiple plant traits and knowledge about the functional characteristics of sphagna is essential for the understanding of their role in natural ecosystems as well as their response to environmental change.

There are differences among the *Sphagnum* species in habitat specialization, growth, and decay. Some are highly specialized, and others can grow across almost the whole range of habitats that the genus occupies (overviews in Flatberg 2013; Rydin and Jeglum 2013). Typically, *Sphagnum* species dominate open bogs, but some are also important in other habitats such as mire margins, poor and rich fens, and forested habitats. The niches of the species differ along gradients of water table and pH. The traits that control niche differentiation should affect the success of individual species.

Many *Sphagnum* species decompose slowly, which results in the accumulation of peat. There are differences in the decay rates of *Sphagnum* species, and previous research has indicated that decay correlates positively with growth (Turetsky et al. 2008; Laing et al. 2014). This variation stems from both the intrinsic decay resistance of individual species and the environment – mainly created by the moss itself – that the species resides in. Generally, hummock species degrade more slowly and hollow species more quickly (Clymo 1965; Johnson and Damman 1991; Belyea 1996; Limpens and Berendse 2003). The wetness of the habitat, as well as the biochemical properties of the species, affects this, but which biochemical properties that contribute the most to decay resistance is unclear (e.g., Verhoeven and Liefveld 1997; Freeman et al. 2001; Hájek et al. 2011). Also, external factors such as aeration of mires because of drought (Freeman et al. 2001) and nitrogen enrichment (Bragazza et al. 2006) have been shown to hamper the growth and increase the decomposition of *Sphagnum*.

The HWT (height above water table), that is, the microtopographical position a species occupies, is one of the most important gradients pointed out as factors driving functional traits in *Sphagnum* (Laing et al. 2014). Hummock species grow in high shoot densities, where smaller pores better retain water and high capillary rise is possible. Subsequently, they are better at avoiding water stress and at the same time at growing far above the water table (Hájek and Beckett 2008; Elumeeva et al. 2011). Hollow species may be more tolerant to desiccation after a slow hardening process (Hájek and Vicherová 2013), but inherent tolerance, as well as recovery of photosynthesis, is still better in hummock species (Hájek and Beckett 2008; Granath et al. 2010). Some of this variation is linked to the different subgenera (sections) of *Sphagnum*, with species of section Acutifolia more related to hummocks and section Cuspidata to hollows. Johnson et al. (2015) have demonstrated a phylogenetic signal in these microhabitat preferences (realized niches), but they concluded that data on the underlying physiological and morphological traits are required to trace the evolution of niches in *Sphagnum*.

Light availability was identified in Laing et al. (2014) as another main gradient driving traits in *Sphagnum*. How biomass production and photosynthesis of *Sphagnum* differ in relation to shade has been studied to a much lesser degree than the relationship between production and water level. In Laing et al. (2014), the most efficient (in terms of photosynthetic nitrogen use efficiency, PNUE) and highest rate of photosynthesis was found in mosses of light‐limited habitats. The open bog species are better adapted to growth in intense light by means of photoprotection (Marschall and Proctor 2004), but CO_2_ diffusion is limited because of thick boundary layers and thin leaves, so the efficiency of their photosynthesis is still very limited. Because of CO_2_ limitation, they face overexcitation and reduce their photosynthesis in favor of less photodamage (Hájek 2014). In conclusion, how light availability affects many functional traits is largely unclear (e.g., Kangas et al. 2014; Laing et al. 2014).

Plant functional types define a group of plants with the same adaptive ecophysiological trait or group of traits, which differentiates that group from others and causes the species within to respond similarly to a change in the environment via the same mechanisms (Smith et al. 1997). Such a species grouping can be used to explain and model ecosystem responses. Generally, the *Sphagnum* species are grouped together as one or a couple of functional types (Lang et al. 2009; Frolking et al. 2010). In reality, they have very different biomass production and decay rates, and therefore different functions in ecosystems. Therefore, species‐specific functional trait values are needed in models that can predict species responses on a global scale (Moor et al. 2015). A functional trait can be described as any character that affects the success of an organism (Reich et al. 2003). The trait can be an adaptive trait that affects, for example, reproduction, colonization, or survival.

We took on a multispecies approach to screen functional traits related to growth and decay in 15 *Sphagnum* species from different sections, vegetation types, degree of shading, and microtopography. Previous studies in this field are limited in their selection of species and commonly investigate only 4–6 species, mainly focusing on the bog expanse. We quantified growth, in terms of production and LI (length increment), and photosynthetic capacity measured as net rate of CO_2_ fixation in standard conditions, and related this to quantified measures of decomposition in field and laboratory conditions.

Specifically, our aims were to:

1. gather trait information as a basis for ecological and environmental research;
2. include species from several *Sphagnum* sections (subgenera) to test whether traits are mostly related to habitat or to phylogeny;
3. test the importance of the wetness and shade gradients in driving species traits, suggested to be the two main environmental gradients in Laing et al. (2014); and
4. test whether the formerly observed trade‐off in *Sphagnum* between growth and decay applies when a wider selection of species and habitats are included. Decay is compared in laboratory and field conditions to assess the intrinsic decay resistance of the litter versus the habitat effects. We test the often‐stated hypothesis of hummock species having a higher intrinsic decay resistance than hollow species.

## Methods

### Species

**Figure 1:**
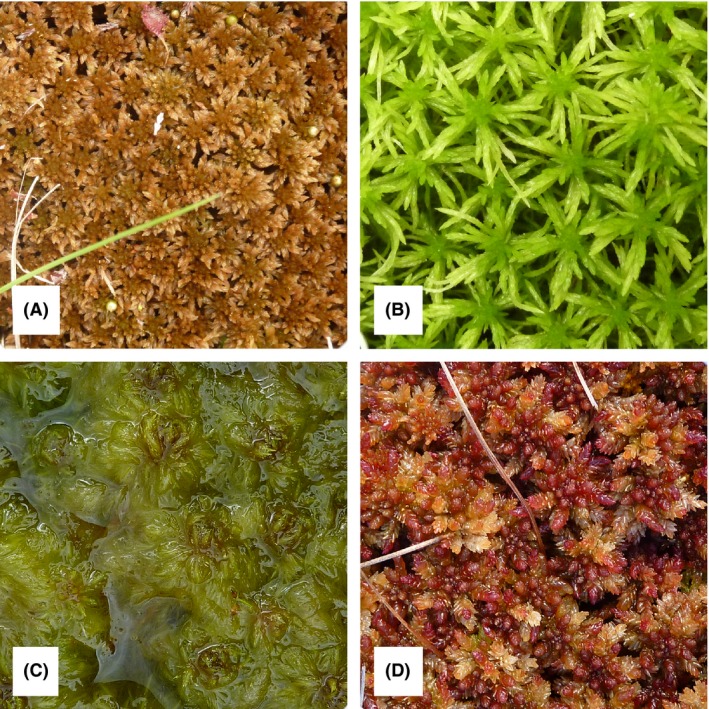
Four of the species included in the study: *Sphagnum fuscum* (A), *S. girgensohnii* (B), *S. cuspidatum* (C), and *S. magellanicum* (D).

Species were chosen to represent different habitats along the bog‐fen gradient and different positions along the microtopographical gradient, with focus on ecologically important species (Fig. 1, Table 1). Microtopographical positions (following Rydin and Jeglum 2013) were used to describe the main niche of the species at the study site, but in the statistical analysis, we use the measured HWT (as described in the sampling section). In general terms, we refer to higher microtopographical levels as hummocks and the lower levels (lawn, carpet, pool) as hollows.

**Table 1:** The study species and their main affinity to microtopographical position, vegetation type, and habitat openness in the study regions. In the analyses, the vegetation types open bog and fen soak were merged into one category (open bog/fen), and lagg fen, mire edge, and spruce forest were merged into another (mire margin). Nomenclature follows Flatberg (2013)

*Sphagnum* species	Section	Author citation	Code	Microtopographical position	Vegetation type	Openness
*S. capillifolium*	Acutifolia	(Ehrh.) Hedw.	CA	Hummock	Pine bog	Semi
*S. fuscum*	Acutifolia	(Schimp.) H. Klinggr.	FU1 FU2	Hummock	FU1 Open bog	Open
					FU2 Rich fen	
*S. girgensohnii*	Acutifolia	Russow	GI	Hummock	Spruce forest	Shaded
*S. rubellum*	Acutifolia	Wilson	RU	Low hummock	Open bog	Open
*S. warnstorfii*	Acutifolia	Russow	WA	Low hummock	Rich fen	Open
*S. angustifolium*	Cuspidata	(Russow) C.E.O. Jensen	AN	Low hummock	Mire edge	Open
*S. balticum*	Cuspidata	(Russow) C.E.O. Jensen	BA	Lawn	Open bog	Open
*S. cuspidatum*	Cuspidata	Hoffm.	CU	Carpet–pool	Open bog	Open
*S. fallax*	Cuspidata	(H.Klinggr.) H. Klinggr.	FA	Lawn	Lagg fen	Open
*S. lindbergii*	Cuspidata	Lindb.	LI	Carpet	Open bog	Open
*S. majus*	Cuspidata	(Russow) C.E.O. Jensen	MJ	Carpet	Open bog	Open
*S. tenellum*	Cuspidata	(Brid.) Brid.	TE	Lawn	Open bog	Open
*S. magellanicum*	Sphagnum	Brid.	MG1	MG1 Lawn–carpet	MG1 Open bog	MG1 open
			MG2	MG2 Hummock	MG2 Pine bog	MG2 semi
			MG3	MG3 Hummock	MG3 Spruce forest	MG3 shaded
*S. papillosum*	Sphagnum	Lindb.	PA	Carpet	Fen soak	Open
*S. contortum*	Subsecunda	Schultz	CO	Lawn	Rich fen	Open

Among the hummock species, *S. capillifolium* grows in the relatively shady wooded marginal slope of the bog (pine bog)*. Sphagnum girgensohnii* is found in heavily shaded spruce forest*. Sphagnum magellanicum* is a species with a wide ecological niche and was sampled from hummocks in both the relatively (pine bog) and the heavily shaded (spruce forest) conditions. *Sphagnum angustifolium* is also a species with a wide niche, but occurs in our sites rather high above the water table. *Sphagnum warnstorfii* is restricted to rich fens. The other hummock species grow in the open bog.

In lawn habitats, we sampled *S. balticum* and *S. tenellum* in the bog, *S. fallax* in the lagg fen surrounding the bog, and *S. contortum* in the rich fen. The wettest growing carpet species in the bog were *S. majus* and *S. cuspidatum* (the latter even extending into bog pools). *Sphagnum lindbergii* and *S. papillosum* grow as soft carpets, the latter in a fen soak (minerotrophic seepage into the bog; Rydin and Jeglum 2013).

Since our main aims were to define strategies for different species and assess the importance of phylogeny versus habitat/strategy, we chose to study the species in their main habitats. However, the mean field approach (using mean trait values per species instead of within‐species trait distribution) has some shortcomings (Violle et al. 2012) and to widen the perspective two species were sampled in different vegetation types and microtopographical positions (Table 1). *Sphagnum magellanicum* is present in quite easily discernable habitats, from different openness and water levels, and was sampled from the two hummock habitats described above and from the open lawn–carpet bog habitat. *Sphagnum fuscum* was sampled from two hummock habitats (open bog, rich fen).

### Study sites

Central to the study was a mire complex in central southern Sweden (Västmanland Province), Kulflyten (59°54′N, 15°50′E). This mire is largely a raised ombrotrophic bog with bog pools and wet fen soaks that are richer in solutes. The outer areas of the bog are pine clad, and a lagg fen of varying width surrounds the bog. Surrounding the mire are young spruce stands; here, the bottom layer primarily consists of *Sphagnum girgensohnii* and common feather mosses. To include rich fen species, we sampled in a small rich fen, Glon (60°31′N, 17°55′E), in the province of Uppland, which is a coastal land uplift area with lime‐rich moraine.

Climate and weather data are summarized in Table 2. Nitrogen deposition is about 0.6 g m^−2^ year^−1^ in both sites (Granath et al. 2009).

**Table 2:** Climate and weather data from the two sites (SMHI 2014)

	Kulflyten	Glon
Mean for 30 years (1982–2013)		
Mean temperature July	16.6°C	16.8°C
Mean temperature December	−2.6°C	−1.0°C
Mean temperature June–September	14.1°C	14.3°C
Mean annual precipitation	733 mm	649 mm
Total precipitation June–September	316 mm	277 mm
2012		
Mean temperature June–September	13.3°C	13.8°C
Total precipitation June–September	459 mm	402 mm
2013		
Mean temperature June–September	14.5°C	14.8°C
Total precipitation June–September	224 mm	260 mm

### Sampling

We chose 143 sampling patches for 13 species at Kulflyten and 18 patches for the three species at Glon. Most of the species have 10 replicates (in total: 161 patches). Each patch was carefully selected so as to be relatively uniform, consisting of close to only one species, with low cover of vascular plants, and typical of that species (Table 1). The replicates were chosen at a minimum distance of 50 m from each other. The patches were big enough for repeated destructive sampling (about 1 m^2^, but often intermingled with smaller parts with other plants), while leaving enough intact *Sphagnum* cover for measuring length growth during two seasons. HWT, that is, the distance between the moss surface and the water table, was measured at each patch (June 2012). The water table position was established in a dip well (perforated pipe, vertically inserted into the peat). HWT was also measured in 2013 but the low precipitation this year resulted in a water table below the installed dip wells at a few patches (>60 cm). Hence, we only use HWT data from 2012, which capture the relative HWT accurately.

The pH was measured (Hanna instruments, HI98129) in surface water in close proximity to the patches in the beginning of the season in 2012 (a few later measurements indicated little variation over the season).

### Length increment and biomass accumulation

At each sampling point, we placed three brush wires for measuring LI (mm). Similar in appearance to a bottlebrush, these small brushes are first inserted into a tight tube open at both ends that keeps the bristles flat and allows inserting the brush into the vegetation without disturbing it much. The brush is then held in place, while the tube is pulled out so that a wire (the brush “handle”) protrudes above the moss surface. The growth is then measured along this wire as a mean of the shoots just surrounding it. For a more comprehensive explanation and illustration, see Rydin and Jeglum (2013). The wires were carefully inserted into the *Sphagnum* vegetation in June (12–20) 2012 and measured again in early October (6–10) to obtain the growth during the vegetation season. In 2013, they were reset in mid‐May (17–20), and LIs were recorded in early October (4–10). LI data were collected as late as we dared to risk snowfall.

The wire method is unreliable in wet carpets that are more unstable and where the shoots do not hold together in an upright position all the time. Here, strings were tied 15 mm from the top of three capitula, and at the end of the season, the new length between top and string was measured.

To calculate production, we needed the mass per unit length of the stem section (Rydin and Jeglum 2013) and number of shoots per unit area. Adjacent to the spots with length measurements, we collected cores of *Sphagnum* of 7 cm diameter. The shoots were counted, and 3‐cm sections below the capitula (defined as the top cm) were collected, thoroughly cleaned from fine roots and alien species, dried to a constant weight (40°C) and weighed. For a few species only 2 cm, and for *S. tenellum* in some cases, only 1‐cm stems were used, because the shoots were already in a forward stage of decomposition further down the stem. The weight of all shoots cm^−1^ in a core divided by the volume of the cylinder per cm gives the dry bulk density (mg cm^−3^). The growth in biomass per individual (G_i_; g) is given by stem mass cm^−1^ × LI, and growth per unit area is G_i_ × shoot density (G_a_; g m^−2^).

### Decay

Shoots were collected at Kulflyten in June (12–20) 2012 and at Glon in July (1–3). The capitula were discarded and 2‐cm shoot sections below the capitula were kept as litter. Stem color was similar but slightly lighter than the capitulum color in all species. A lighter color indicates less pigments and this part of the plant can be considered semi‐active. It has been shown that photosynthesis can occur, but there is a net carbon loss under light conditions (Wallén et al. 1988; Laing et al. 2014). The litter was cleaned from liverworts, roots, and rhizomes, then dried at 60°C, and approximately 100 mg per sample was put into a nylon mesh bag (mesh size 300 *μ*m). Decay rates were measured in the field and laboratory to be able to separate the effect of litter quality and habitat conditions. Half of the litterbags were buried at the origin on the mire during July 9–15 about 5 cm below the moss surface. The other half were incubated in the laboratory starting 10 September 2012, in separate 15 × 15 cm containers, and partly covered with a plastic film to avoid evaporation, but still allowing air to enter. They were placed in a dark room with a constant environment in an inoculum containing nutrients, deionized water, and microorganisms extracted from peat (following Hájek et al. 2011). The water was topped up when starting to dry out and the litter bags were moved around inside the containers every couple of weeks to increase oxygen availability.

After 7 months, the laboratory litterbags were dried and weighed to obtain mass loss until this point. They were put back in the inoculum April 28, and dried and weighed again after another 7 months. This was done to get an appreciation of when the bulk of the decay happens, and to discover whether decomposition continued after the first 7 months. The field litterbags were collected in late September 2013, dried at 60°C, and weighed to get the decomposition over 14 months, including almost two full vegetation seasons.

### Photosynthetic capacity

Net CO_2_ fixation rate was used as a measure for photosynthetic rate. An IRGA (infrared gas analyzer, PP Systems) was connected to a specially designed set of chambers, using one chamber for moss and one as a reference. Air was taken from the outside of the building and passed through the empty reference chamber or the moss chamber. The moss CO_2_ uptake is the difference between the reference and the moss chamber. The ambient CO_2_ concentration was between 360 and 380 ppm. The flow rate was 100 mL min^−1^, and the temperature was kept around 25°C. Light was provided from a glasshouse lamp (cool white, 4500 K) and was kept between 330 and 530 *μ*mol m^−2^ sec^−1^, these light conditions being above or close to light saturation, but below photoinhibition (Harley et al. 1989; Marschall and Proctor 2004).

The samples were collected in 7‐cm cores in the field 5–6 September 2012. They were kept in the garden for acclimation for at least 5 days, well hydrated and not exposed to direct sunshine, and measurements were carried out within 2 weeks of sampling (11–20 September). Because the measurements of CO_2_ exchange rate are time‐consuming, we measured five replicates from each species in random order. To process all 161 samples would have forced us to keep the cores in the garden for another couple of weeks.

Samples that were to be measured were moved into the laboratory (glasshouse) at least 30 min prior to measurements. Capitula were then cut and placed moistened onto a net in the chamber (diameter 5 cm) close together, but not compressed. The number of capitula varied between 10 and 30 depending on the size of the species. Following the procedure of Granath et al. (2009), we monitored gas exchange while the mosses slowly dried out and recorded the maximum net CO_2_ fixation rate. Dry weight was obtained after the capitula were dried at 60°C. Optimum water content for photosynthesis has been reported to be between 500 and 3000%, while the theoretical optimum is much lower (170–240%) (Hájek 2014). In our measurements, the average is in the lower end of the measured range: The lowest average was 650% (*S. capillifolium*) and the highest 1211% (*S. magellanicum* from pine bog habitats).

The calculated rate of maximum net CO_2_ fixation at optimal light and water is defined as the photosynthetic capacity, expressed based on an individual shoot (NP_i_; mg h^−1^), dry weight (NP_g_; mg g^−1^ h^−1^), or area (NP_a_; mg cm^−2^ h^−1^), following calculations in Granath et al. (2009).

### Carbon and nitrogen concentration

Litter was collected in October 2013. Each collected sample of litter was milled to a fine powder and carbon and nitrogen concentrations (C/N ratio) were measured with a Costech ECS 4010.

### Statistical analyses

We used linear regressions and PCA to test relationships between measured variables. Residuals were checked for normality and data were log‐transformed when required. ANOVA and Tukey tests were used to analyze the relationships between PCA scores and openness and linear regression for the relationship with HWT. We used the statistical software R 3.2.1 (R Core Team, 2015). The NIPALS algorithm (Dray and Dufour 2007) was used for PCA so that rows with missing data entries could be used.

The phylogenetic influence was tested using *Sphagnum* section as a fixed factor. These sections are, indeed, well separated in the *Sphagnum* phylogeny (Johnson et al. 2015). However, to investigate the phylogenetic effect on trait distribution (i.e., using the first two axes of the PCA as response variables) in *Sphagnum,* we deepened our analyses and used the phylogenetic tree of the 15 species used in our study. The phylogenetic tree was created by extracting the best tree from 1000 posterior *Sphagnum* trees containing 41 species (Johnson et al. 2014, 2015) using treeAnnotator (Drummond and Rambaut 2007). This tree was then trimmed down to the 15 species of interest and plotted using the R packages “ape” and “phytools” (Fig. S1) (Paradis et al. 2004; Revell 2012).

To quantify the relative contribution of phylogeny, habitat (vegetation type, shade, HWT), species, within species (same species but different vegetation type) and within sample (i.e., the combination of measurement error and variation in replicate patches nested in vegetation type) to PC1 and PC2, we first applied a linear mixed model using the R package nlme (Pinheiro et al. 2015). In the analyses, the vegetation types open bog and fen soak were merged into “open bog/fen”, and lagg fen, mire edge, and spruce forest were merged into “mire margin” (cf. Rydin and Jeglum 2013). In three separate models, vegetation type, shade, and HWT were fitted as fixed effects and the two sampling levels (species, within species) as nested random effects and the residual variance is within‐sample variation. PC1 and PC2 were response variables and separate models were run for each predictor (intercept (no predictor), shade, vegetation type, HWT). Species‐level BLUPs (*n* = 15) were extracted from these models and used as the response variable in PGLS (phylogenetic generalized least square) models where the *Sphagnum* tree is included as covariance matrix and a parameter (Pagel's *λ*, Pagel 1999) estimates to what extent the shared evolutionary history (as approximated by Brownian motion) can account for the observed trait distribution. From the PGLS models, the phylogenetic variance (*V*_phy_, *λ *= *V*_phy_/(*V*_phy_ + *V*_error_)) can be extracted and compared with the other variance components. The amount of variation explained by the fixed effect was calculated following Nakagawa and Schielzeth (2013). We only fitted models where the phylogenetic effect was estimated after accounting for the fixed effect due to computational difficulties of such complex mixed models. Our analyses are not robust tests of phylogenetic signal or models of evolution; they are rather an approximate quantification of sources of variation along our trait axes.

## Results

### Stand structure and environment

The results from the measurements of stand structure and HWT are summarized in Table 3. Shoot density ranged between 1 cm^−2^ (*S. contortum*) and 9 cm^−2^ (*S. tenellum*), and dry bulk density ranged between 3 mg cm^−3^ (*S. contortum*) and 20 mg cm^−3^ (*S. fuscum*, bog).

**Table 3:** Summarized stand structure variables and the HWT (height above water table) at which the studied species grew in 2012, showing mean ± SE for the n patches sampled

Species	Code	Dry bulk density (mg cm^−3^)		Shoot density (cm^−2^)		HWT 2012 (mm)	*n*
		2012	2013	2012	2013		
Acutifolia							
* S. capillifolium*	CA	12.9 ± 0.92	7.1 ± 0.84	3.4 ± 0.18	2.7 ± 0.22	377 ± 49.8	10
*S. fuscum,* bog	FU1	13.8 ± 1.05	20.4 ± 2.58	5.2 ± 0.39	7.1 ± 0.7	267 ± 14.4	10
*S. fuscum*, fen	FU2	7.4 ± 1.15	7.6 ± 1.7	3.4 ± 0.37	3.4 ± 0.65	272 ± 40.5	6
*S. girgensohnii*	GI	4.4 ± 0.44	4.1 ± 0.52	1.3 ± 0.1	1.2 ± 0.05	278 ± 43.3	9
*S. rubellum*	RU	17.3 ± 0.83	17 ± 2.04	5.6 ± 0.37	6.7 ± 0.7	136 ± 13.7	10
*S. warnstorfii*	WA	6.1 ± 0.76	5.1 ± 0.47	2.1 ± 0.28	2.3 ± 0.17	206 ± 29.4	6
Cuspidata							
*S. angustifolium*	AN	8.2 ± 0.55	5.8 ± 0.55	2.9 ± 0.12	2.9 ± 0.24	127 ± 10.1	10
*S. balticum*	BA	16 ± 1.49	11.9 ± 1.06	2.6 ± 0.29	3.4 ± 0.38	54 ± 6.3	9
*S. cuspidatum*	CU	7.8 ± 0.62	6.2 ± 0.45	1.6 ± 0.09	1.5 ± 0.18	3 ± 2.1	10
*S. fallax*	FA	6.4 ± 0.65	6.4 ± 0.69	2.3 ± 0.12	1.8 ± 0.09	114 ± 5.7	10
*S. lindbergii*	LI	18.7 ± 3.32	9.4 ± 1.03	1.5 ± 0.17	1.2 ± 0.12	41 ± 6.6	10
*S. majus*	MJ	9.6 ± 0.84	9.4 ± 0.85	1.4 ± 0.11	1.8 ± 0.23	19 ± 3	9
*S. tenellum*	TE	18.5 ± 1.15	17.4 ± 1.54	9.2 ± 0.89	7.5 ± 0.77	52 ± 8.4	9
Sphagnum							
*S. magellanicum*, open bog	MG1	13.7 ± 1.33	6.9 ± 0.67	1.5 ± 0.09	1.3 ± 0.11	54 ± 6.2	10
*S. magellanicum*, pine bog	MG2	10.8 ± 0.85	6.5 ± 0.6	1.7 ± 0.09	1.3 ± 0.08	335 ± 29.5	10
*S. magellanicum,* spruce forest	MG3	6.3 ± 1.71	3.9 ± 0.72	1.1 ± 0.07	0.8 ± 0.07	304 ± 27.3	7
*S. papillosum*	PA	13.4 ± 1.63	8.3 ± 0.97	1.3 ± 0.09	1.2 ± 0.08	57 ± 9.1	10
Subsecunda							
*S. contortum*	CO	4.8 ± 0.31	3.2 ± 0.21	1.1 ± 0.11	1 ± 0.09	78 ± 14.6	6

Precipitation (monthly average) differed clearly between the 2 years, which was reflected in higher HWT in 2013 for all plots, except for the fen plots. The biggest effect may have been on *S. cuspidatum*, which in June 2012 had its capitula growing close to the water level, while in 2013, they grew around 13 cm above the water table.

The pH was around 3.8 in the bog plots and 6.4 in the fen plots in 2012 and in 2013 slightly higher for the bog plots (4.4–4.5) and lower in the fen (5.5–6.0).

### Growth

**Figure 2:**
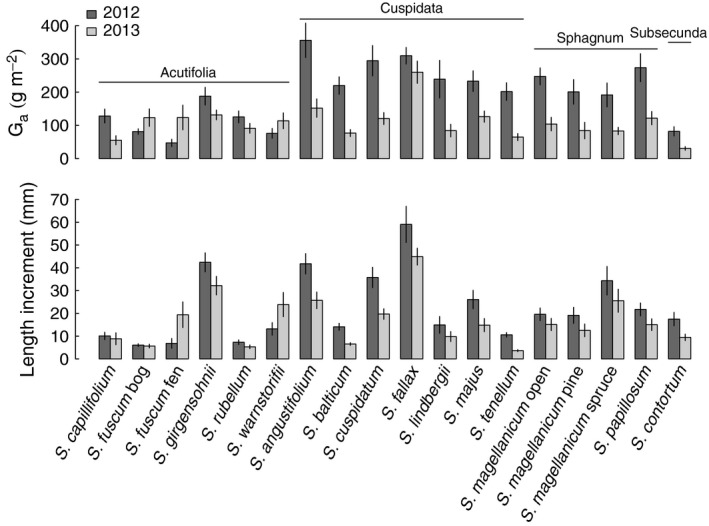
Upper panel shows growth in biomass G_a_ (g m^−2^) and lower panel length increment (mm) in two successive years (*n* = 6–10) for the sampled *Sphagnum* species. Bars show mean ± SE.

In the wet year, 2012, the growth in biomass (G_a_) was generally higher in sections Cuspidata and Sphagnum than in Acutifolia and Subsecunda (Fig. 2). This tendency is less clear for LI where *S. girgensohnii* and *S. warnstorfii* (Acutifolia) grew at least as much as *S. balticum*,* S. lindbergii*, and *S. tenellum* (Cuspidata).

**Figure 3:**
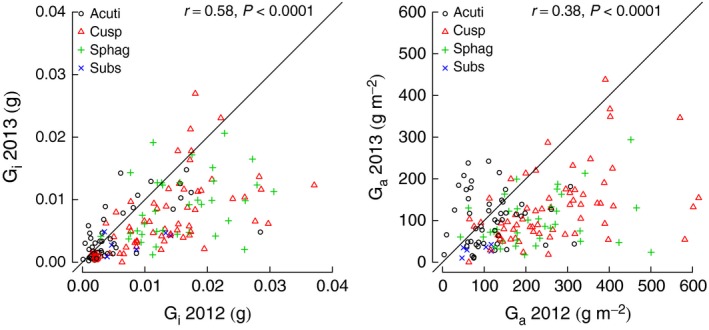
Relationships between biomass growth in 2012 and 2013 per shoot (left; *n* = 156) and per area (right; *n* = 155) with lines for 1:1 relationship.

Most patches had a faster biomass accumulation in the wet 2012 than in the dry 2013 (points below the 1:1 line in Fig. 3). The difference between growth in the two years is relatively small in section Acutifolia (Fig. 2), and the slow‐growing *S. fuscum* and *S. warnstorfii* even grew more in 2013 (Figs. 2, 3). Hence, the differences between sections were largely evened out in 2013. To test for the effect of section, we made a multiple regression with growth in 2012 and section (categorical) as predictors for growth in 2013. For G_a_ (but not for G_i_), there was a significant effect of section (Adj. *R* ^2^ = 0.17 for the combined effect of growth in 2012 and section, *P* < 0.0001, df = 150) with a significant difference between Subsecunda (Fig. 3 right: all points below the 1:1 line) and Acutifolia (most points above the line).

*Sphagnum girgensohnii* is an aberrant species in section Acutifolia, growing considerably more in length and slightly more in biomass than other Acutifolia species, and similar to the Cuspidata species, it grew more in 2012 than in 2013 (Fig. 2).

The variation in biomass growth G_i_ was partly explained by HWT when all species were included in the model (aim 3) (regression: *P* < 0.0001; *R* ^2^ = 0.11; df = 151). However, there was no support for a negative effect of HWT on growth when only one species (*S. magellanicum*, sampled in three vegetation types with patches ranging in HWT between 20 and 570 mm) was analyzed (*P* = 0.15; df = 23). The effect of HWT on LI was small (*P* = 0.007; *R* ^2^ = 0.05; df = 152).

### Decomposition

As expected, field decomposition was negatively affected by increasing wetness of the habitat (aim 3) (regression: *P* < 0.0001; *R* ^2^ = 0.18; df = 151).

**Figure 4:**
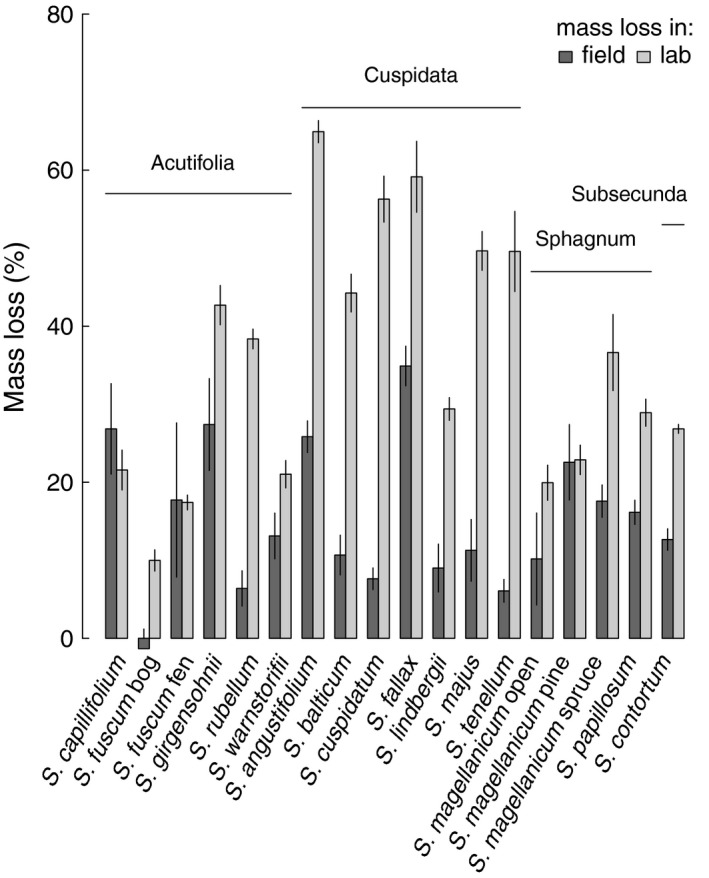
Decomposition measured as mass loss after 14 months in the field and in the laboratory (*n* = 6–10). Bars show mean ± SE.

In the laboratory experiment, species from section Cuspidata lost more in decay than the other sections (Fig. 4), exceptions being *S. lindbergii* (low decay; sect. Cuspidata) and *S. girgensohnii* and *S. rubellum* (high decay; sect. Acutifolia). There was substantial variation among species laboratory decay from only 10% mass loss in bog‐inhabiting *S. fuscum* to about 65% mass loss in *S. angustifolium*. The field decay varied from −1% in *S. fuscum* bog samples (i.e., no measurable decomposition) to 35% in *S. fallax* (Fig. 4).

**Figure 5:**
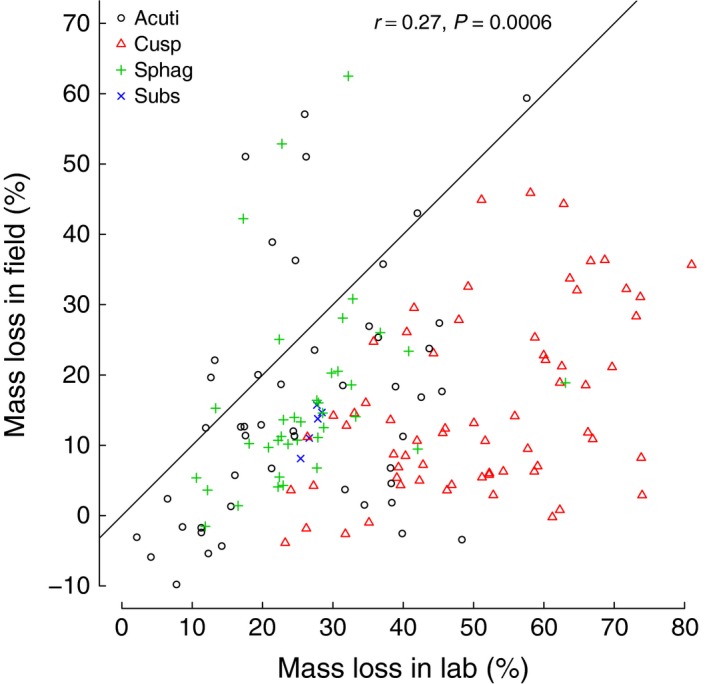
The relationship between decay in field and in laboratory after 14 months measured as % mass loss of litter; *n* = 155. The line shows the 1:1 relationship.

Mass loss from litter was generally higher in the laboratory (Figs. 4, 5). All Cuspidata patches lost more in the laboratory than in the field, while a few Acutifolia and Sphagnum patches showed the opposite (Fig. 5). The decay in the field and the laboratory was correlated (*P* = 0.0006; *r* = 0.27). We performed regressions with laboratory decay as a predictor for field decay, the rational being that laboratory decay should reflect the inherent species properties and field decay would also include environmental effects (regression: *P* = 0.0006; R^2^ = 0.074; df = 153). Including section as a factor in the regression (*P* < 0.0001; Adj. *R* ^2^ = 0.13; df = 150) shows that decay was significantly lower in samples of section Acutifolia (average ± SE (%)) field: 15.2 ± 2.5; laboratory: 25.5 ± 1.9) than in Cuspidata (field: 15.5 ± 1.6; laboratory: 50.6 ± 1.8).

The results after 7 and 14 months of decomposition under laboratory conditions were similar, and measurements at the two times correlated well (*P* < 0.0001; *r* = 0.93). The samples that decomposed faster in the first period also decomposed faster in the second period.

### Photosynthetic capacity

**Figure 6:**
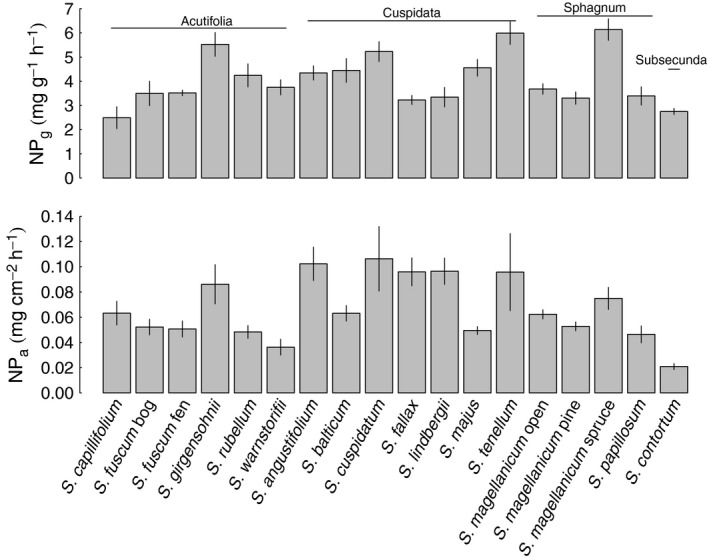
Upper panel: The photosynthetic capacity (net rate of CO_2_ fixation under standard conditions) expressed per unit dry mass (NP_g_: mg g^−1^ h^−1^; *n* = 5). Lower panel: per unit area (NP_a_: mg cm^−2^ h^−1^; *n* = 5; bars show mean ± SE).

The photosynthetic capacity expressed per unit mass (NP_g_) and per unit area (NP_a_) varied as shown in Figure 6. On mass basis, the highest values were in *S. tenellum* together with the forest patches of *S. girgensohnii* and *S. magellanicum*. The highest NP_a_ values were found in several Cuspidata species (*S. angustifolium*,* S. cuspidatum*,* S. fallax*,* S. lindbergii*, and *S. tenellum*) and in *S. girgensohnii*. Naturally, species with larger capitula (such as the ones in our forest patches, and *S. fallax* and *S. cuspidatum*) had higher NP_i_ (data not shown).

The photosynthetic capacity on a mass basis (NP_g_) was higher in heavily shaded habitats compared to open habitats (aim 3), both overall (ANOVA: *F*_2,91_ = 18.17; *P* < 0.0001) and within a species (*S. magellanicum*) (ANOVA: *F*_2,13_ = 23.47; *P* < 0.0001). However, we could not see this difference on the area‐based measurement (NP_a_).

### Interrelationships among attributes

**Figure 7:**
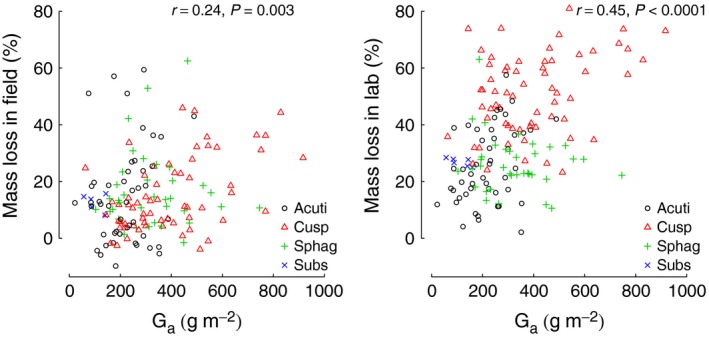
Relationships between decay rate in the field (left; *n* = 150) or in the laboratory (right; *n* = 148) and the growth in biomass on an area basis G_a_ (total g m^−2^ for 2012 and 2013).

Overall, our data show a positive relationship between growth and decay, supporting the hypothesis of a trade‐off between these two traits (aim 4) (Fig. 7). The regression model(s), using the pooled G_a_ for 2012 and 2013 to predict decay, explained a small amount of the variation for field decay (*P* = 0.003; *R* ^2^ = 0.060; df = 148). Including sections as a factor did not improve the model for field mass loss (*P* = 0.02; Adj. *R* ^2^ = 0.055; df = 145). Slightly more of the variation was explained for laboratory mass loss (*P* < 0.0001; *R* ^2^ = 0.20; df = 151), especially when including section as a factor (*P* < 0.0001; Adj. *R* ^2^ = 0.51; df = 148). Species with higher biomass accumulation have higher decomposition rates in laboratory conditions, and they more commonly belong to section Cuspidata.

**Figure 8:**
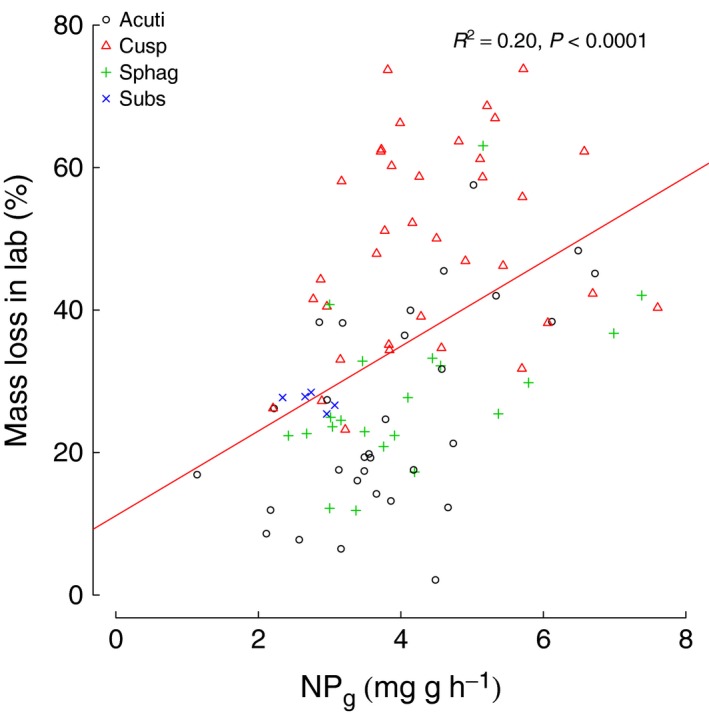
The mass loss in laboratory conditions as a function of the photosynthetic capacity (net rate of CO_2_ fixation under standard conditions); *n* = 93.

The positive relationship between NP_g_ and mass loss in laboratory also supported the trade‐off (Fig. 8; regression: *P* < 0.0001; *R* ^2^ = 0.20; df = 91). Again, adding section increases explanatory power (*P* < 0.0001; Adj. *R* ^2^ = 0.53; df = 88). Patches of section Cuspidata have mainly positive residuals and patches from section Acutifolia negative residuals.

**Figure 9:**
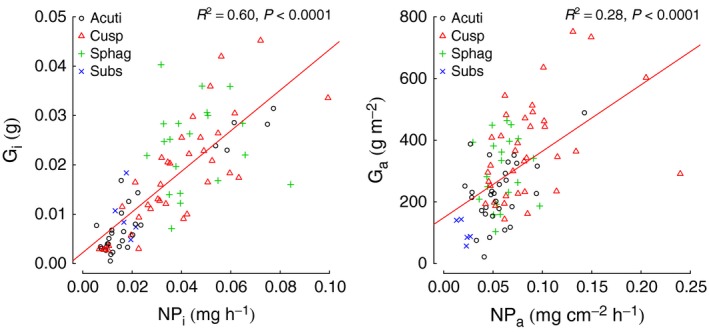
Growth in biomass (pooled 2012 and 2013) as a function of photosynthetic capacity (net rate of CO_2_ fixation under standard conditions) per individual (left; *n* = 90) and per area (right; *n* = 90).

We tested photosynthetic capacity (NP) as predictor for biomass accumulation in the field (G). For the shoot‐based measurements, there was a relatively strong relationship (Fig. 9, left; *P* < 0.0001; *R* ^2^ = 0.60; df = 88), but with negligible effect of section included as factor (*P* < 0.0001; Adj. *R* ^2^ = 0.61; df = 85). The relationship was weaker on an area basis (Fig. 9, right; *P* < 0.0001; *R* ^2^ = 0.28; df = 88), but here there was an effect of section (*P* < 0.0001; Adj. *R* ^2^ = 0.35; df = 85). Section Cuspidata had a higher G_a_ and NP_a_ and was significantly different from Acutifolia.

**Figure 10:**
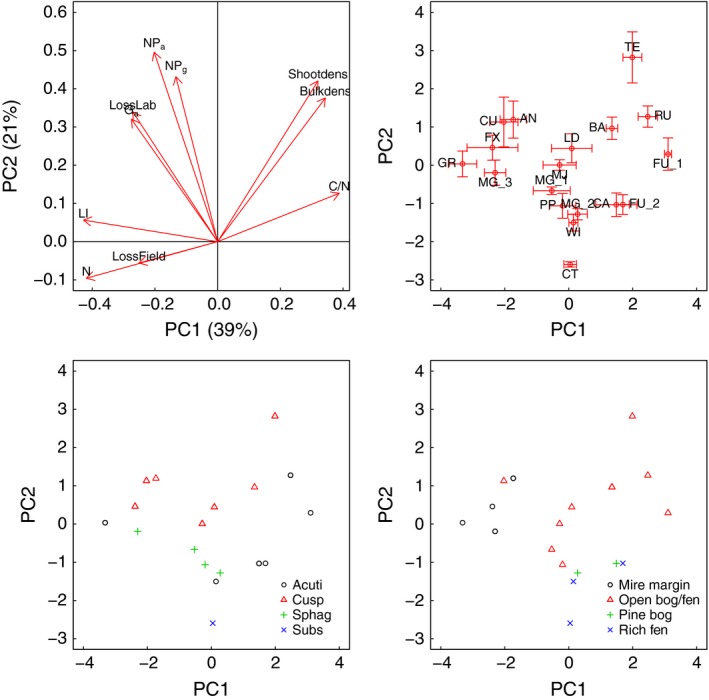
PCA using the NIPALS algorithm (Dray and Dufour 2007). Top left: Trait space showing the factor loadings of the variables: Shootdens = average shoot density between 2012 and 2013; Bulkdens = average bulk density between 2012 and 2013; C/N ratio of litter; N = nitrogen concentration of litter; LossField = mass loss during two seasons (%); LI = pooled length increment in 2012 and 2013 (mm); G_a_ = pooled biomass growth (g m^−2^) 2012 and 2013; LossLab = mass loss of litter in laboratory after 14 months (%); NP = photosynthetic capacity (net rate of CO_2_ fixation under standard conditions) expressed per unit dry mass (NP_i_; mg g^−1^ h^−1^); and per unit area (NP_a_; mg cm^−2^ h^−1^). Out of the variance explained by the three first axes, PC1 explains 39% and PC2 21%. Top right shows the species distribution along the PC axes (mean ± SE). Bottom left shows the species grouped by *Sphagnum* section, and bottom right shows the species grouped by vegetation type.

The principal components analysis showed that the first axis (explaining 39% of the total variation) was positively associated with C/N ratio, bulk, and shoot density, and negatively with mass loss in field, N, and LI. For the second component (21%), the strongest influence came from the NP variables, area‐based biomass growth (G_a_), and mass loss in laboratory (Fig. 10). The variables for N concentration and field mass loss are closely related and are, together with LI and C/N ratio, the strongest contributors to PC1. Also laboratory decay and biomass accumulation are closely related, but vary both along PC1 and PC2.

When plotting species based on phylogeny (sections; Fig. 10, lower left), most Acutifolia species group in the direction of low growth, low decay, and high C/N. The exception is *S. girgensohnii*. The Cuspidata species are held together in the upper part of the diagram (high growth, high laboratory decay), and the Sphagnum section clusters in the central part. When species are plotted based on habitat (vegetation type), *S. girgensohnii* clearly groups with the other mire margin species (Fig. 10, lower right).

**Figure 11:**
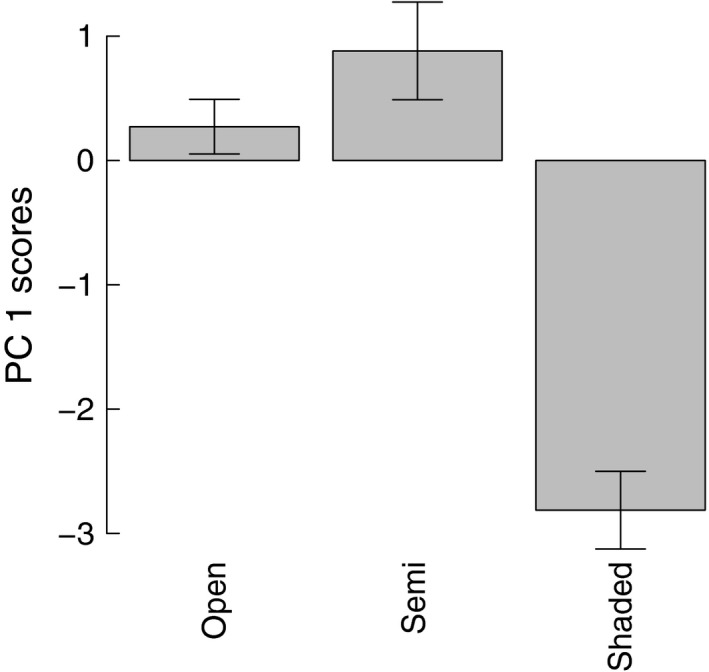
PC1 scores for species representing different habitats (mean ± SE). There was an overall significant effect of openness of the habitat (ANOVA_2,91_: *F* = 14.55; *P* < 0.0001), open and shaded differ (Tukey: *P* < 0.0001), and semi‐open and shaded differ (Tukey: *P* < 0.0001).

Openness and wetness have been suggested to be important factors driving traits in *Sphagnum* (Laing et al. 2014). For habitat openness, we found a significant relationship with PC1 (Fig. 11, ANOVA: *F*_2,91_ = 14.18; *P* < 0.0001). Species growing well in shade are differentiated from the ones growing on the open bog and in semi‐open habitats (Tukey: *P* < 0.0001 for both comparisons). Including the phylogenetic structure in the analysis shows that shade explains 17% of the variation along PC1, while the phylogeny accounts for 6%, species 36%, within species 14%, and within sample (species nested in vegetation type) 27% of the variation. The same analyses using vegetation type as a predictor instead of shade produce the corresponding numbers: vegetation type 32%, phylogeny 10%, species 26%, within species 7%, and within sample 26%. For habitat wetness, we found a weak negative relationship between HWT and PC2 (regression: *P* = 0.0018; *R* ^2^ = 0.11; df = 88).

Adding the phylogenetic structure in the analysis shows that phylogeny accounted for more variation in PC2 (18–26%) compared to PC1 (6–10%). However, along PC2, there is a strong covariation with vegetation type making it hard to disentangle the two factors. Having vegetation type as a predictor (explaining 20%) decreases the phylogenetic effect from 29% to 18%. For both axes, within‐species variation and within‐sample variation amount to about 31–65% of the total variation depending on the fitted model.

## Discussion

We provide trait values regarding stand structure, growth, production, and decay of 15 *Sphagnum* species (aim 1). To some extent, our results support the previously found trade‐off between growth and decay (Figs. 7, 10; Turetsky et al. 2008; Laing et al. 2014), where hollow species grow and decay faster than hummock species. However, previous research has compared few species, mostly from open bogs. As we broaden this by including a wider range of species and habitats, a more complex picture emerges with intriguing effects of vegetation type, microhabitat, and phylogeny, and we discuss further based on aims 2–4.

### Stand structure, growth, and photosynthetic capacity

Photosynthetic capacity (Fig. 6) reflects the species potential photosynthesis, whereas biomass accumulation and LI also reflect the environment over the season. Broadly speaking these two growth measures gave similar responses among species, but deviations from that pattern are of interest.

Many hollow species grew faster than hummock species (aim 3), as has been reported in earlier studies that examined only a few species (reviewed in Gunnarsson 2005; Rydin et al. 2006). But in the wet year, in which the species could be expected to grow near their potential, two species with wide niches, *S. angustifolium* (sect. Cuspidata) and *S. magellanicum* (sect. Sphagnum), grew more than other hummock species. As a result, all species from sections Cuspidata and Sphagnum grew more than those from sections Acutifolia and Subsecunda. This indicates that phylogeny is more important than microhabitat in explaining potential productivity.

Also for LI, hummock species are in general slow‐growing. *S. girgensohnii* is an example of a hummock species with fast LI, which could be explained by the shaded habitat that promotes elongation but hampers photosynthesis and biomass accumulation. Among the hollow species, *S. tenellum* (sect. Cuspidata) made little length growth. In the PCA, LI is only weakly related to production (G_a_) and may therefore not be a good response measure in environmental research if different species or habitats are compared.

The innate growth rate and the photosynthetic capacity also showed a higher average in sect. Cuspidata, but the variation within the section is large. Species with larger capitula had higher capacity and biomass accumulation per shoot. This effect of size is less important when calculated per unit area (Fig. 7, right panel) due to allometric constraints where shoot mass scales as the inverse of numerical density (Harley et al. 1989; Laing et al. 2014). The shade‐inhabiting *S. girgensohnii* stands out in section Acutifolia as a fast photosynthesizer. Also from the spruce forest, *S. magellanicum* had a markedly higher rate (especially per unit dry weight), compared to other *S. magellanicum* habitats and other species in section Sphagnum. Laing et al. (2014) proposed the hypothesis that photosynthetic capacity and length growth are higher in shade growing species, but biomass produced is not necessarily higher than in open habitats. Overall, our data give some support for this hypothesis, especially within *S. magellanicum*. It would be interesting to investigate whether this is an acclimation or adaptation to be efficient under a tree canopy. Unlike the other species in section Acutifolia, *Sphagnum girgensohnii* lacks red pigments suggested to have a function in photoprotection (Bonnett et al. 2010), and it is noteworthy that the forest samples of *S. magellanicum* also lacked the red pigmentation so typical of this species. Bryophytes are generally shade tolerant, but sphagna growing in open habitats seem generally less so (Marschall and Proctor 2004). As red and brown species lose a lot of these pigments in shade, a possible mechanism is reallocation of nitrogen from photoprotective pigments to chlorophyll and rubisco. However, in these shaded habitats, the high photosynthetic capacity is combined with high length growth, but does not lead to very high biomass growth.

### Decomposition

We made laboratory experiments to assess the intrinsic decay resistance of the litter and field experiments to also include the habitat effects. Here, one should bear in mind that the habitat is created by the species and is therefore also a functional trait that can be seen as a reflection of the extended phenotype (Dawkins 1982). Decay resistance can be enhanced by leaching of organochemical substances which slow down decomposition (Verhoeven and Toth 1995) and by differential capillarity and water‐holding capacity of the *Sphagnum* species that waterlogs the habitat. Waterlogging, in turn, affects aeration and redox potential through many hydrological feedbacks (Waddington et al. 2015).

Decomposition in the laboratory was relatively fast during the first 7 months compared to the following 7 months, and the time chosen to measure decay may therefore affect the results. However, the relative decomposition of species was consistent, as in Johnson and Damman's (1991) field decay study, and measurements after 7 and 14 months were highly correlated. Therefore, we feel confident that our results capture the relative decomposition relationships among species.

Several studies found that hollow species decompose faster than hummock species (Clymo 1965; Johnson and Damman 1991; Belyea 1996; Limpens and Berendse 2003). As predicted, we found that species from section Cuspidata, that is, mainly hollow species, generally decay faster than section Acutifolia species where we find most hummock species. This is especially true in laboratory conditions. Reciprocal litter bag experiments have suggested that the decomposition is less dependent on the mire habitat where they are degrading than on the species (Turetsky et al. 2008). Our results indicate that this is an oversimplification. With our intention to test whether traits are related mostly to habitat or phylogeny (aim 2), we conclude that there is a higher intrinsic resistance to decay in most Acutifolia species. In contrast, there are greater habitat constraints by wetness in Cuspidata species, as well as in *S. rubellum* (the wettest growing Acutifolia species) and *S. magellanicum* (in its wet bog habitat).

Generally, *Sphagnum* litter decomposes more slowly than vascular plant litter (review in Scheffer et al. 2001), but our results show that variation among *Sphagnum* species should be taken into account in ecological research. Cornwell et al. (2008) performed a metastudy on vascular plants and found large differences in decay between functional groups (18‐fold), but we found large differences within the genus *Sphagnum* (6.5‐fold for laboratory comparison and 15‐fold for field, comparing species averages).

### Trade‐offs, phylogeny, and environments

Both the regression models testing the relationship between growth (G and NP) and decomposition and the PCA illustrate the complexity of trade‐offs between traits related to growth and decay (aim 4).

The PCA illustrates relationships and trade‐offs among traits. Nitrogen concentration has been pointed out as a main factor influencing decay rate in *Sphagnum* (Lang et al. 2009) and is here seen to be a main contributor in the PCA. Nitrogen concentration could be seen as a result of fast growth and is strongly related to length growth, but length growth does not always reflect the biomass gained. Nitrogen concentration and length growth may also be positively affected by nitrogen concentration in the surroundings, and it is likely that the mire margin sites have more available nitrogen than the bog (Bragazza et al. 2005). However, the PCA indicates that high nitrogen content in bog species also leads to higher rates of decay and production.

The decay resistance in *Sphagnum* is often attributed to higher concentration of polysaccharides (Hájek et al. 2011) or phenolics (Verhoeven and Liefveld 1997; Freeman et al. 2001). It has been suggested that there is a resource allocation trade‐off in *Sphagnum* between structural and metabolic carbohydrates (Turetsky et al. 2008). This implies that faster‐growing species invest in easily degradable carbohydrates, which leads to the expected faster decomposition in hollow species. Support for this hypothesis was reported by Laing et al. (2014). Our results support the direction of this trade‐off, but the relationships are rather weak. Our next step must be to measure the organochemical compounds in the very same samples as used in this paper, to further test the mechanisms behind the trade‐off.

The distribution of the species in the trait space shows how phylogeny (sections) and habitat (vegetation type) to various degree cluster in the trait space. HWT and shade have been suggested to be the two most important variables in structuring the functional traits in *Sphagnum* (Hájek et al. 2009; Laing et al. 2014). In support of previous studies, light availability was associated with PC1 and the traits driving this separation in the trait space were LI and nitrogen concentration (Figs. 10, 11). The heavily shaded patches differ markedly from the others, and this is reflected in the PCA. The shaded habitat may induce greener shoots with a higher capacity for photosynthesis, and competition for light may trigger length growth and elongation. While most of the total variation along PC1 can be explained by vegetation type and species (58%), there is some evidence that phylogeny plays a role (10%). The large proportion of variance explained by within species (between vegetation types) and within sample (measurement error and species variation nested in vegetation type) highlights the importance of extended sampling in trait sampling campaigns and the dangers of a species mean‐centered approach (Albert et al. 2010). The contrasting behavior between the most shaded *S. girgensohnii* and *S. magellanicum* and their open habitat relatives illustrates this.

Wetness, or HWT, was a less strong explanatory variable for the multivariate trait space than we expected (cf. Laing et al. (2014)) and was only weakly correlated with PC2. The variables for photosynthetic capacity (NP_g_ and NP_a_) are mainly driving the 2nd axis in the PCA, suggesting that HWT controls the photosynthetic capacity to some extent. The phylogenetic effect may, however, be a much more important component along PC2, but covariation with vegetation type makes it hard to draw firm conclusions. The indications of a phylogenetic effect on trait space are in line with Johnson et al. (2015) who reported a strong phylogenetic signal in habitat (microtopography and shade) preference in *Sphagnum*.

### Between‐year variation

In our study, we were able to compare the traits in two contrasting years, one exceptionally wet and one dry. Due to the stochastic nature of droughts, such events are rarely investigated in natural habitats even though they are ecologically important: Gerdol et al. (2008) found *Sphagnum* production to be around 50% lower in a dry year compared to a normal year.

Potentially, the higher rate of decomposition in Cuspidata species could lead to an ever‐increasing amount of Acutifolia hummock peat. However, this is not the case. In fact, the structures of hummocks and hollows have been found to be very stable (Belyea and Clymo 2001). The microtopographical pattern we observed can be caused by the dry year leading to a larger decrease in growth and increase in decomposition in Cuspidata than in Acutifolia species. This is likely to be the explanation behind the relative microtopographical stability; a fluctuating weather and climate will benefit both strategies but under opposite situations (dry vs. wet year). Many species, whether they are typical of hummocks or hollows, grow as well or better in hollows compared to in hummocks, but only hummock species can last on hummocks. Basically, hummock species avoid desiccation (by capillary water transport) rather than tolerating it (references in Rydin et al. 2006). Our results show that species from Acutifolia have less biomass accumulation than Cuspidata species in a year with good water availability. But in a dry year, only hummock species (i.e., mostly Acutifolia) can retain enough water for photosynthesis and growth (Fig. 3). For example, *Sphagnum fuscum* grew about as much in length and biomass in both years (in the fen even more in the dry year). There is evidence that even though hollow species are more susceptible to desiccation, they may be more tolerant (Hájek and Vicherová 2013), at least if hardening is induced slowly. In 2013, the drying out was probably too fast and too severe. This indicates that desiccation avoidance is more important than induced desiccation tolerance. However, *S. fallax* with among the highest growth in both years was not severely affected by the dry spell. This result is difficult to explain; it had some shade in the lagg, but other shaded species were severely affected in the dry year.

There should be a similar effect on decomposition in dry versus wet years, that is, decay should be low in hollows in wet years and high in dry years when they are not waterlogged. For hummock species, the water content may not change that much in the upper part of the hummock even though the water table was lowered due to capillary rise (water table depth – moss productivity feedback (Waddington et al. 2015)). In a dry period, the looser upper layers of the hollows cannot sustain a hydrological connection with the water table and will face desiccation rapidly. This feedback will promote decomposition, thereby making the peat denser in drier years. Here, we cannot separate the effects of the dry and the wet years, but it seems that the effect of the drought was not severe enough for the hollows to reach the same decay rate as in the laboratory. The differences between species between years indicate, as formerly suggested in Rice et al. (2008), that there is a trade‐off in *Sphagnum* between tolerating environmental stress and fast biomass production. Our results also highlight the importance of considering temporal variation in traits (Violle et al. 2012).

### Variation within species

The *S. magellanicum* patches from the pine bog and open bog had similar intrinsic decay resistance. We interpret the lower field decay in the open bog as an effect of habitat – the species grow much closer to the water table in the open bog and consequently will be less aerated. In contrast, the forest samples had lower intrinsic decay resistance. While this too could be a habitat effect, it has recently been suggested that mire margin populations of *S. magellanicum* may differ genetically (perhaps even taxonomically) from bog populations (Kyrkjeeide et al. 2016). Open, ombrotrophic bog is the main habitat of *S. fuscum*, and the low decay in bog compared to fen is compatible with this. With similar biomass growth, the potential to form hummocks seems smaller in the fen. So, even if traits are relatively constant within species, we should expect them to be modified when species grow outside their main habitat. Reciprocal transplants of the litter would be necessary to disentangle the role of genetic variation versus plasticity.

## Conclusions

Functional traits will be key features for predictions in current environmental research such as earth system modeling (Wullschleger et al. 2014) and species distribution models (Moor et al. 2015). In terms of peatlands, this includes how changes in species composition or the environment can alter growth and decomposition and hence carbon sequestration. Our study provides a comprehensive starting point for a *Sphagnum* trait data base.

Litter quality can be seen as an intrinsic property of the species. However, some species are more affected by the environmental conditions they grow in (and which they create themselves). Particularly, habitat wetness strongly reduces the realized rate of decomposition in hollow inhabiting species. Further studies on the biochemistry of *Sphagnum* are necessary to make progress in our understanding of the underlying causes for long‐term slow decay.

We found support for the formerly observed trade‐off between growth and decay, but the relationships are constrained by phylogeny and habitat. This suggests that large‐scale carbon flux models ought to use species‐specific parameters rather than treating *Sphagnum* as one functional type, or as a matter of routine lump species into sections or categorize them as hummock or hollow species. We found that it is important to distinguish between traits that represent innate qualities (such as photosynthesis and decomposition under laboratory conditions) and those that represent effects of habitat on *Sphagnum* and effects of *Sphagnum* on habitat.

Species trait combinations are, as expected, linked to the wetness gradient (HWT), and this relationship is to a large degree linked to phylogeny. Traits are also related to shading, and here there seems to be a strong habitat effect in addition to a phylogenetic one.

## Data Accessibility

Data available from the Dryad Digital Repository, http://dx.doi.org/10.5061/dryad.62054

## Conflict of Interest

None declared.

## Supporting information


**Figure S1.** The phylogenetic tree of the 15 species used in our study. The phylogenetic tree was created by extracting the best tree from 1000 posterior *Sphagnum* trees containing 41 species (Johnson et al. 2015) and then trimmed down to the 15 species of interest.
