# Supplementary figures and images for: Photosynthesis, growth, and decay traits in Sphagnum – a multispecies comparison
Source: Ecol Evol. 2016 Apr 12;6(10):3325–41. doi: 10.1002/ece3.2119 (PMC4833502; doi:10.1002/ece3.2119)

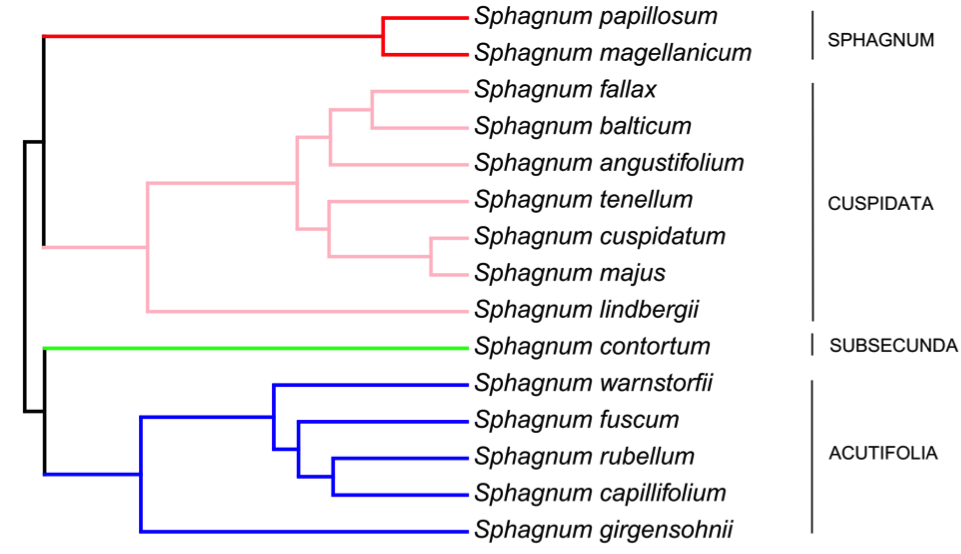

Supplement: Supplementary file 1 — Figure S1. The phylogenetic tree of the 15 species used in our study. The phylogenetic tree was created by extracting the best tree from 1000 posterior Sphagnum trees containing 41 species (Johnson et al. 2015) and then trimmed down to the 15 species of interest. [file ECE3-6-3325-s001.png]
